# Biological Signal Processing and Analysis for Healthcare Monitoring

**DOI:** 10.3390/s22145341

**Published:** 2022-07-18

**Authors:** Yunfeng Wu, Behnaz Ghoraani

**Affiliations:** 1 School of Informatics, Xiamen University, 422 Si Ming South Road, Xiamen 361005, China; 2 Department of Computer and Electrical Engineering and Computer Science, Florida Atlantic University, Boca Raton, FL 33431, USA; bghoraani@fau.edu

Nowadays, portable and wireless wearable sensors have been commonly incorporated into the signal acquisition modules of healthcare monitoring systems. These multi-modal wearable sensors are able to simultaneously acquire multi-channel physiological signals from the human body, which may support the functions of point-of-care pathology screening and accurate diagnostic decision making in healthcare monitoring systems [1]. Practical applications call for advanced signal processing and computational intelligence techniques that may explore the intrinsic features of biological signals and provide useful diagnostic information.

The motivation of this Special Issue is to collect the recent development of biological signal processing and analysis methods, and their applications in healthcare monitoring and computer-aided diagnosis. The biological signal processing and analysis techniques contain the design of signal preprocessing tools for portable wearable sensors, artifact cancellation methods for signal quality improvement, nonlinear analysis for the representation of signal complexity or dynamics, feature extraction using time-frequency analysis or statistical models, pattern classifications, and computer-aided diagnosis based on deep learning neural networks or other computational algorithms, along with well-devised healthcare monitoring systems for clinical applications.

Wearable or implantable sensors can record different biological signals generated by complex physiological processes. The combination of sensor arrays is a promising solution for the design of effective healthcare monitoring systems. In this Special Issue, we strive to highlight the state-of-the-art signal processing technologies that are suited for multi-modal physiological data integration and fusion to generate comprehensive and clinically actionable information. The body–seat interface temperature measurement system developed by Liu et al. [2] demonstrates the integration of temperature and infrared sensor data for a long-term healthcare monitoring application.

This Special Issue covers the topics of biomedical signal feature extractions using the temporal waveform analysis, frequency analysis, time-frequency analysis, and nonlinear analysis. Biological signals commonly exhibit different spatiotemporal morphology styles in their waveforms. For example, electrocardiogram (ECG) signals present quasi-periodic QRS peaks, which can be detected for RR interval estimation and heart rate variability analysis. Time-domain waveform processing procedures may include temporal signal segmentation, signal decomposition and reconstruction, envelop extraction, and so forth. The rhythm and periodicity properties of biomedical signals can be extracted and measured in the frequency domain using the fast Fourier transform (FFT), discrete Fourier transform, Hilbert transform, and wavelet transform techniques [3]. The short-time Fourier transform, wavelet transform, and time-frequency analysis based on matching pursuit decomposition can provide more details about the time-varying frequency distributions for signal approximation in order to study the time-varying frequency properties of nonstationary signals [4]. The work of Zong and Wu [5] is an interesting biomedical signal frequency analysis study that investigates the relationship between wideband and narrowband simulation noise with various center frequencies in the dynamic process of auditory perception with lower-level transient memory of acoustic features.

Recently, nonlinear analysis methods, such as fractal dimension analysis and signal complexity analysis, have been used for the multiscale dynamics representation of physiological signals and the interpretation of nonstationary biological process. Fractal dimension parameters are often used to quantify the self-similarity of biological signals at different scales. Signal complexity can be represented as the level of randomness or fluctuations in the time domain. Entropy models are the appropriate statistical techniques to study the nonlinear dynamics of nonstationary biomedical signals with different model parameters [6]. This Special Issue is expected to address the new progress of the entropy measures such as approximate entropy, sample entropy, fuzzy entropy, and permutation entropy for the analysis of biomedical signal dynamics or complexity.

Computational machine learning methods such as supervised learning, unsupervised learning, semi-supervised learning, reinforcement learning, and ensemble learning algorithms have been frequently employed in healthcare monitoring applications and medical diagnosis decision-making systems. Powered by the emerging cloud-computing technologies and hardware infrastructures, deep learning paradigms have been extensively applied in biomedical research projects and engineering applications [7]. The state-of-the-art deep learning neural networks have the advantages of generating localization details of the region of interest and mapping the spatial features at different levels in the multiple network layers using encoding and decoding structures. The most prevailing deep learning architectures include convolutional neural networks, fully convolutional networks, generative adversarial networks, and recurrent neural networks. In this Special Issue, we strive to highlight the recent development of deep learning neural networks in the applications of physiological data fusion, medical image segmentation [8], lesion detection, texture analysis, and image registration.

## Data Availability

Not applicable.
